# Characterization of distinct sub-cellular location of transglutaminase type II: changes in intracellular distribution in physiological and pathological states

**DOI:** 10.1007/s00441-014-1990-x

**Published:** 2014-09-11

**Authors:** Mauro Piacentini, Manuela D’Eletto, Maria Grazia Farrace, Carlo Rodolfo, Franca Del Nonno, Giuseppe Ippolito, Laura Falasca

**Affiliations:** 1 Department of Biology, University of Rome ‘Tor Vergata’, Rome, Italy; 2 Laboratory of Electron Microscopy, National Institute for Infectious Diseases I.R.C.C.S. ‘L. Spallanzani’, Via Portuense 292, Rome, Italy; 3 Department of Pathology, National Institute for Infectious Diseases I.R.C.C.S. ‘L. Spallanzani’, Rome, Italy; 4 Epidemiology and Pre-Clinical Research Department, National Institute for Infectious Diseases “L. Spallanzani”, Rome, Italy

**Keywords:** Transglutaminase 2, Mitochondria, Nuclear localization, Endoplasmic reticulum, Cell surface distribution, Human

## Abstract

Transglutaminase type II (TG2) is a pleiotropic enzyme that exhibits various activities unrelated to its originally identified functions. Apart from post-translational modifications of proteins (peculiar to the transglutaminase family enzymes), TG2 is involved in diverse biological functions, including cell death, signaling, cytoskeleton rearrangements, displaying enzymatic activities, G-protein and non-enzymatic biological functions. It is involved in a variety of human diseases such as celiac disease, diabetes, neurodegenerative diseases, inflammatory disorders and cancer. Regulatory mechanisms might exist through which cells control multifunctional protein expression as a function of their sub-cellular localization. The definition of the tissue and cellular distribution of such proteins is important for the determination of their function(s). We investigate the sub-cellular localization of TG2 by confocal and immunoelectron microscopy techniques in order to gain an understanding of its properties. The culture conditions of human sarcoma cells (2fTGH cells), human embryonic kidney cells (HEK293^TG^) and human neuroblastoma cells (SK-n-BE(2)) are modulated to induce various stimuli. Human tissue samples of myocardium and gut mucosa (diseased and healthy) are also analyzed. Immuno-gold labeling indicates that TG2 is localized in the nucleus, mitochondria and endoplasmic reticulum under physiological conditions but that this is not a stable association, since different locations or different amounts of TG2 can be observed depending on stress stimuli or the state of activity of the cell. We describe a possible unrecognized location of TG2. Our findings thus provide useful insights regarding the functions and regulation of this pleiotropic enzyme.

## Introduction

Transglutaminase type II (TG2) is expressed ubiquitously and abundantly and has been implicated in a variety of cellular processes, such as differentiation, cell death, inflammation, cell migration and wound healing (reviewed in Fesus and Piacentini 2002; Lorand and Graham 2003; Fésüs and Szondy 2005; Collighan and Griffin 2009). It seems to exert contradictory activities, including both pro- and anti-apoptotic functions (Fésüs and Szondy 2005) and has been shown to be involved in the maturation of autophagolysosomes (D'Eletto et al. 2009) indicating that TG2 plays a role between apoptosis induction and autophagy (Fimia and Piacentini 2010).

Aberrant activation of TG2 or deregulation of its function(s) is involved in a variety of human diseases, such as celiac disease, diabetes, neurodegenerative diseases, multiple sclerosis and rheumatoid arthritis (Facchiano et al. 2006). A role in inflammatory disorders and septic shock has also been shown (Falasca et al. 2005, 2008). Moreover, multiple studies have revealed elevated TG2 expression in many types of cancer cells (Mehta et al. 2010).

To explain this plethora of observations, several points have been considered such as the cell type investigated (TG2 is particularly abundant in endothelial cells, fibroblasts and monocytes/macrophages), the different experimental conditions and stimuli utilized (Ca^++^, nucleotides, nitric oxide, reactive oxygen species) and the distinct protein-protein interactions in the local microenvironment, all of which might regulate the activities of TG2 (Jeong et al. 2009). The sub-cellular localization of TG2 has been suggested as another important determinant of its functions. TG2 is believed to create distinct molecular interactions in the different sub-cellular compartments, which in turn exert diverse effects on cellular physiology. Depending on its localization, TG2 presents various biochemical activities, such as a transglutaminase (TGase), G protein (Gh), kinase, protein disulfide isomerase (PDI) and/or as an adaptor protein (Park et al. 2010). At low calcium concentrations, under normal physiological conditions, intracellular TG2 displays no TGase activity, although it displays other activities including those of Gh, kinase and PDI. TG2 is predominantly a cytoplasmic protein, although it is also found in the nucleus and mitochondria, on the plasma membrane, in the extracellular cell surface and in the extracellular matrix (ECM). Extracellular TG2 is involved in wound healing and scarring, tissue fibrosis and metastatic cancer (Aeschlimann and Thomazy 2000). At the cell surface, TG2 has also been implicated in cell adhesion as an adaptor protein and in ECM remodeling via TGase activity (Bergamini et al. 2011). TG2 has been shown to translocate dynamically depending on the state of cell proliferation or in response to the elevation of intracellular calcium concentrations (Korner et al. 1989; Ientile et al. 2007).

Knowledge of the sub-cellular localization of TG2 has greatly expanded recently but studies in the field primarily rely on the biochemical analysis of fractionated samples. However, for a faithful demonstration of enzyme sub-cellular location, electron microscopy is the most accurate technique in that it allows the visualization of morphological events at high resolution. In this study, TG2 was studied by the immuno-gold technique in order to investigate, at the electron microscopy level, the presence of the protein in diverse sub-cellular compartments. TG2 ultrastructural localization was analyzed in the context of a variety of cell types and tissues and under diverse conditions (physiological or non-physiological). The data obtained provide useful clues and insights with regard to the way in which TG2 expression is modulated in response to various stimuli.

## Materials and methods

### Cells and tissue samples

Various cell lines have been used to determine the intracellular localization of TG2 and the way that enzyme expression is modulated in response to diverse conditions.

The cell line 2fTGH (purchased from Cancer Research Technology, London, UK) was derived from HT1080 human sarcoma cells (Rani et al. 1996) and has been previously established to express high TG2 levels (D'Eletto et al. 2012).

HEK293^TG^ (human embryonic kidney) cells were stably transfected with active wild-type TG2 as described previously (Rossin et al. 2012). In brief, HEK293 cells (purchased from American Type Culture Collection, Rockville, Mass., USA) were transfected with the full-length human TG2 gene inserted into the pLPCX vector by using Lipofectamine 2000 (Invitrogen, Life Technologies, Grand Island, N.Y., USA) according to the manufacturer's instructions and were then selected for puromycin (Sigma-Aldrich, St. Louis, Mo., USA) resistance by using the antibiotic added to the culture medium (2 μg/ml).

SK-n-BE(2) neuroblastoma cells (purchased from American Type Culture Collection) were used to provide further insight into current thinking regarding the involvement of TG2 in the development of drug resistance in cancer cells.

Cells were cultured in Dulbecco's modified Eagle's medium (Invitrogen, Life Technologies) supplemented with 10 % fetal bovine serum, 2 mM L-glutamine, 100 mg/ml streptomycin and 100 units/ml penicillin (Sigma-Aldrich) in a 5 % CO_2_ incubator.

Human tissue samples, including intestinal and heart biopsies, were used as “in vivo” models.

### Confocal microscopy

The 2fTGH cells were fixed in ice-cold ethanol/acetone (1:1) at −20 °C for 10 min. Samples were briefly rinsed in phosphate-buffered saline (PBS), permeabilized with 0.1 % Triton X-100 in PBS for 5 min and blocked with 1 % bovine serum albumin (BSA) and 10 % normal goat serum in PBS for 30 min. Primary antibodies, namely mouse anti-TG2 (CUB 7402, Thermo Scientific, Rockford, Ill., USA), rabbit anti-calreticulin antibody (Stressgen, Enzo Life Sciences, Farmingdale, N.Y., USA), rabbit anti-p62/SQSTM1 (MBL, Woburn, Mass., USA) and rabbit anti-Tom20 antibody (Santa Cruz Biotechnology, Dallas, Texas, USA), were incubated for 1 h with the cells at room temperature. After being washed, the cells were incubated with Alexa488- or Alexa594-fluorochrome-coupled secondary antibodies directed against rabbit or mouse (Molecular Probes, Life Technologies, Grand Island, N.Y., USA).

Coverslips were mounted in SlowFade-Anti-Fade (Invitrogen, Life Technologies). Fluorescence was analyzed with a TCS SP2 confocal laser scanning microscope (Leica Microsystems, Wetzlar, Germany). Digital images obtained separately in both channels through a 63× objective (zoom factor 2×) were acquired with Leica Confocal Software.

### Processing for electron microscopy

Cells or tissue samples were fixed with 4 % formaldehyde (prepared fresh from paraformaldehyde) in 0.1 M sodium cacodylate buffer (pH 7.4) containing 2.5 % sucrose for 2 h at 4 °C. After being washed in sodium cacodylate buffer and following dehydration in a series of increasing concentrations of ethanol (70 % ethanol for 20 min; 95 % ethanol, two changes for 20 min), samples were infiltrated with a mixture of LR-White resin (Agar Scientific, Stansted, Essex, UK) and 95 % ethanol (1:l) for 2 h and with pure LR-White overnight at 4 °C. Samples embedded into gelatin capsules filled with LR-White were then transferred into an oven at 55 °C for 24 h to polymerize.

### Immunoelectron microscopy

For immunolabeling, ultrathin sections (60 nm thickness) were washed three times with PBS and three times with PBS containing 1 % BSA and 0.15 % glycine, followed by a 30-min blocking step with 5 % normal goat serum. Samples were incubated with the anti-TG2 (CUB 7402) diluted 1:25 for 1 h at room temperature. Where indicated, an anti-calreticulin antibody (Stressgen) was used. After being washed in PBS, samples were incubated with appropriate secondary antibody conjugated to 15-nm or 5-nm gold particles (BioCell, Cardiff, UK). Sections were stained with 2 % uranyl acetate and observed under a Zeiss EM900 transmission electron microscope. Images were captured digitally with a Mega View II digital camera (SIS, Soft Imaging System, Münster, Germany).

### Controls for immunostaining

The specificity of the immunoreaction was assessed in all cases by omitting the primary antibodies from the labeling protocol and incubating the sections only in the protein-gold-conjugated secondary antibodies

### Quantitative evaluation of labeling density

Quantitation of TG2 within enterocytes was assessed by counting the numbers of gold particles per area of sectioned microvilli. Fifty random digital electron microscope images of the apical part of enterocytes (at a magnification of 20,000×) were taken for each group of patients examined (3 celiac patients and 3 patients with celiac disease in remission). The area of microvilli and the number of gold particles were measured on the images by using image-processing software (analySIS 3.1; SIS), making it possible to draw a region of interest (ROI) containing the brush border or a part of the cytoplasm of individual cells. The density of gold labeling was calculated and the differences between samples were statistically evaluated by the Student's *t*-test.

## Results

### TG2 location inside the cell under normal conditions

**Fig. 1:**
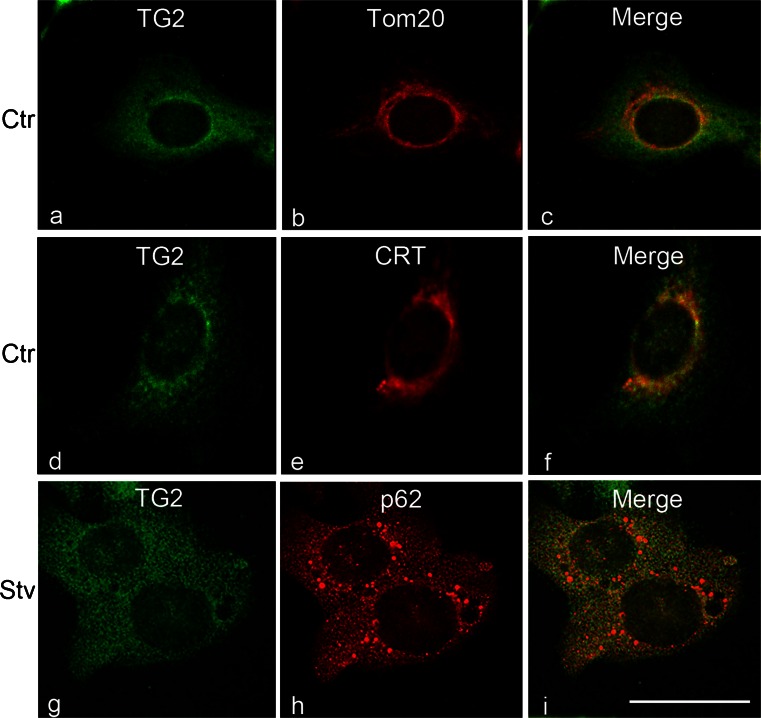
Confocal microscopy analysis of transglutaminase type II (TG2) distribution in 2fTGH cells. Cells cultured under normal conditions (*Ctr*) or subjected to starvation (*Stv*) were stained with anti-TG2 (**a**, **d**, **g**, *green*); with Tom20 (**b**, *red*), a marker of mitochondria; with anti-calreticulin (**e**, *CRT*, *red*), a marker of endoplasmic reticulum; or with anti-p62 (**h**, *red*) localizing to autophagosomes. TG2 was distributed in the cytoplasm with a granular pattern. The merged image of the double-fluorescence signals (*Merge*) highlights the presence of TG2 on mitochondria (**c**) and endoplasmic reticulum (**f**) in controls and on autophagic vesicles (**i**) during autophagy induction. *Bar* 6 μm

Immunofluorescence microscopy of TG2 distribution revealed a diffuse punctate labeling pattern inside the cells (Fig. 1a, d). Double-immunofluorescence of TG2 with antibodies directed against proteins specific for mitochondria (Fig. 1b) and endoplasmic reticulum (ER; Fig. 1e) showed that TG2 was sporadically associated with these organelles (Fig. 1c, f). To characterize the nature of this widespread localization inside the cell further, we performed a detailed study of the presence of TG2 in various cell compartments by immunoelectron microscopy.

Results obtained in HEK293^TG^ cells, under normal culture conditions, showed that the enzyme was localized at several sites inside the cell. However, it never appeared interspersed in the cytoplasm but was always associated with a cell structure, such as the nucleus, mitochondria, or plasma membrane.

**Fig. 2:**
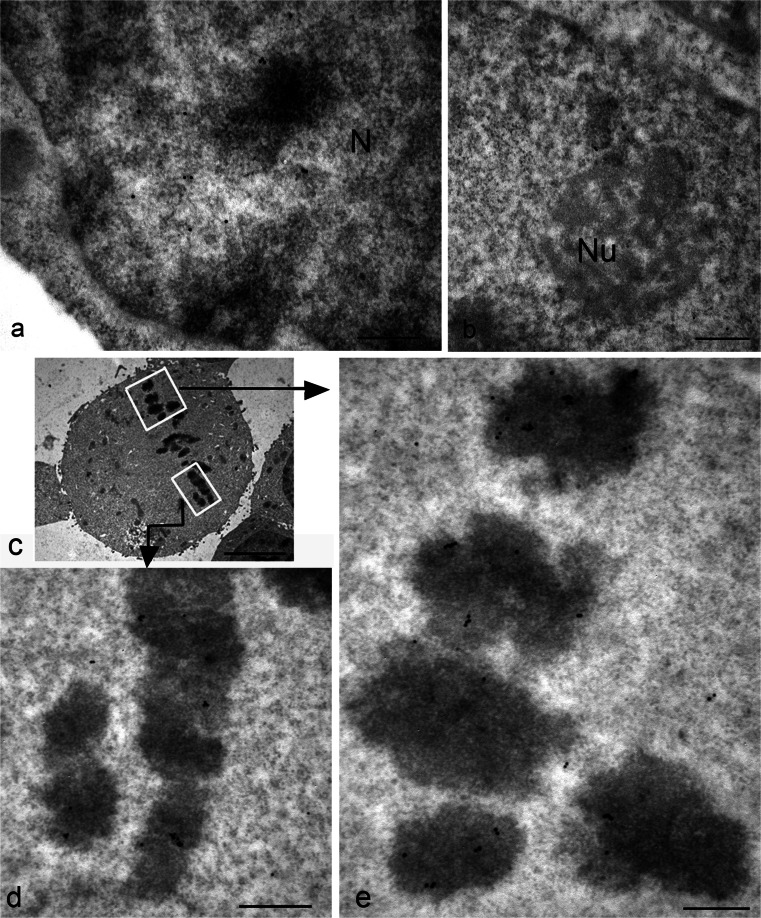
Immuno-gold detection of TG2 in the nucleus of HEK293^TG^ cells shows positive labeling. **a** During interphase, gold granules are localized over the euchromatin regions of the nucleus (*N*), whereas heterochromatin are devoid of labeling. **b** No particles are present at the level of the nucleolus (*Nu*). **c** General view showing the chromosomes gathered at the metaphase plate. **d**, **e** Higher magnification images showing details of the condensed chromatin clearly labeled with clusters of gold granules. *Bars* 0.3 μm (**a**, **b**, **d**, **e**), 3 μm (**c**)

**Fig. 3:**
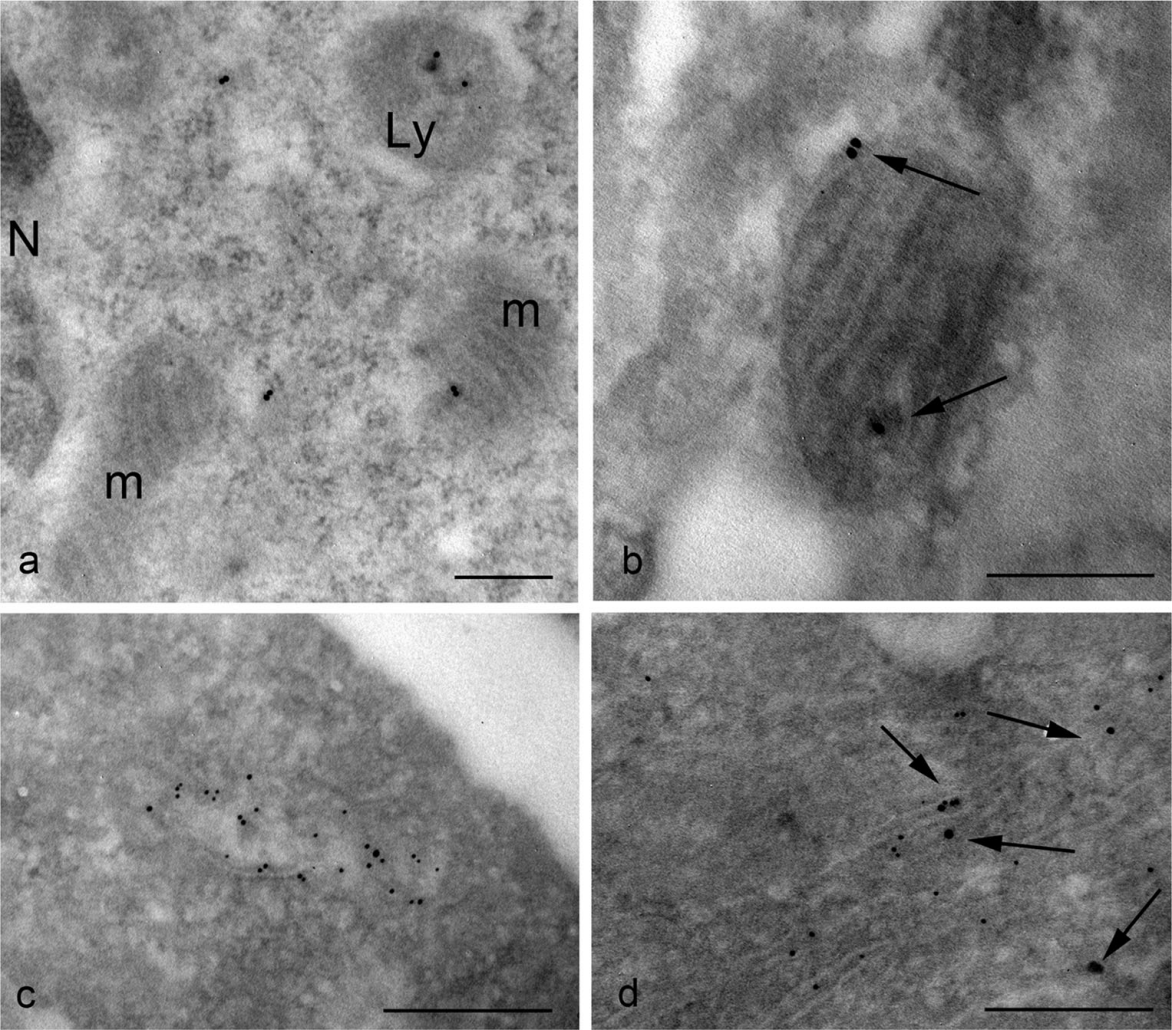
Ultrastructural detection of TG2 in HEK293^TG^ cells shows distinct organelle localization of the enzyme. **a** Electron micrograph of a cytoplasmic region containing two mitochondria (*m*) exhibiting negative or faint staining and a lysosome (*Ly*) that is clearly labeled. **b** Mitochondrion displaying gold granules associated with both the outer membrane and the matrix inside the organelle. **c** Numerous gold particles reveal that TG2 is localized to the endoplasmic reticulum (ER) compartment. **d** Double-immunolabeling with specific antibodies against TG2 (*arrows*) and calreticulin (*small gold granules*) shows a positive reaction on the ER cisternae. *Bars* 0.3 μm

The presence of TG2 in the nucleus has been reported by others in various cell types, although no one has previously demonstrated with which nuclear structure (nuclear membrane, nucleoplasm, nucleolus, chromatin) it is associated. Gold particles unequivocally revealed the presence of the enzyme as being mostly associated with euchromatin (Fig. 2a), confirming the notion that nuclear TG2 especially impacts on the regulation of gene expression via post-translational modification of and/or interaction with transcriptional factors and related proteins. No particles are present at the level of nucleolus (Fig. 2b); this indicates that, although polyamine conjugates have been reported to be localized within the nucleolus, the enzyme is normally not associated with this organelle. Interestingly, during mitosis when transcription is inhibited, TG2 was still associated with chromatin but, in this case, it appeared specifically localized on condensed regions of chromosome (Fig. 2c). Although not a typical mitochondrial protein, approximately half of the cell content of TG2 has been reported to be associated with mitochondria (Rodolfo et al. 2004). Nevertheless, the immuno-gold localization showed that, under normal basal conditions, TG2 was rarely found on mitochondria (Fig. 3a, b); the enzyme seemed to be associated with both the outer mitochondrial membrane and with the matrix (Fig. 3b). A cell compartment that was not previously considered to be a site of localization for TG2 was the ER. Our observations showed positive immunoreaction associated with the ER (Fig. 3c, d). In order to establish the nature of the membranous structures under consideration, we performed double-immunoreactions for calreticulin, a classic chaperone-like ER resident and found that TG2 staining strongly overlapped with calreticulin immunoreactivity (Fig. 3c, d).

**Fig. 4:**
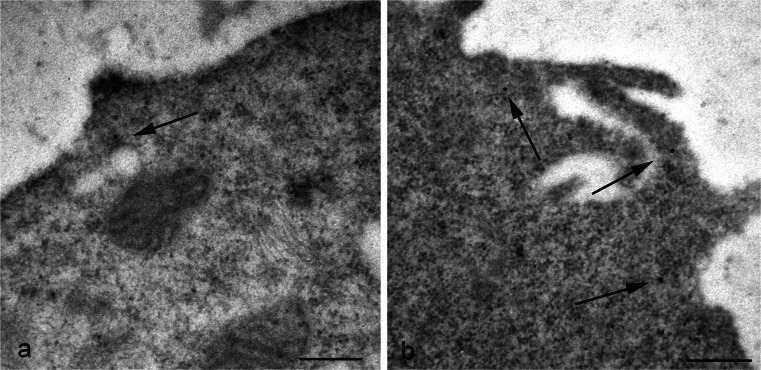
Representative electron micrographs showing cell membrane detection of TG2. **a** Surface of the cells is usually weakly labeled. **b** Gold particles are preferentially found at the level of cell membrane projections (*arrows*). *Bars* 0.3 μm

Concerning the cell surface, our immuno-gold analysis of TG2 demonstrated a low density of gold particles on the plasma membrane of the cells under normal conditions (Fig. 4). In addition, the label indicated that, when present, the enzyme was preferentially located on cell surface projections (Fig. 4).

**Fig. 5:**
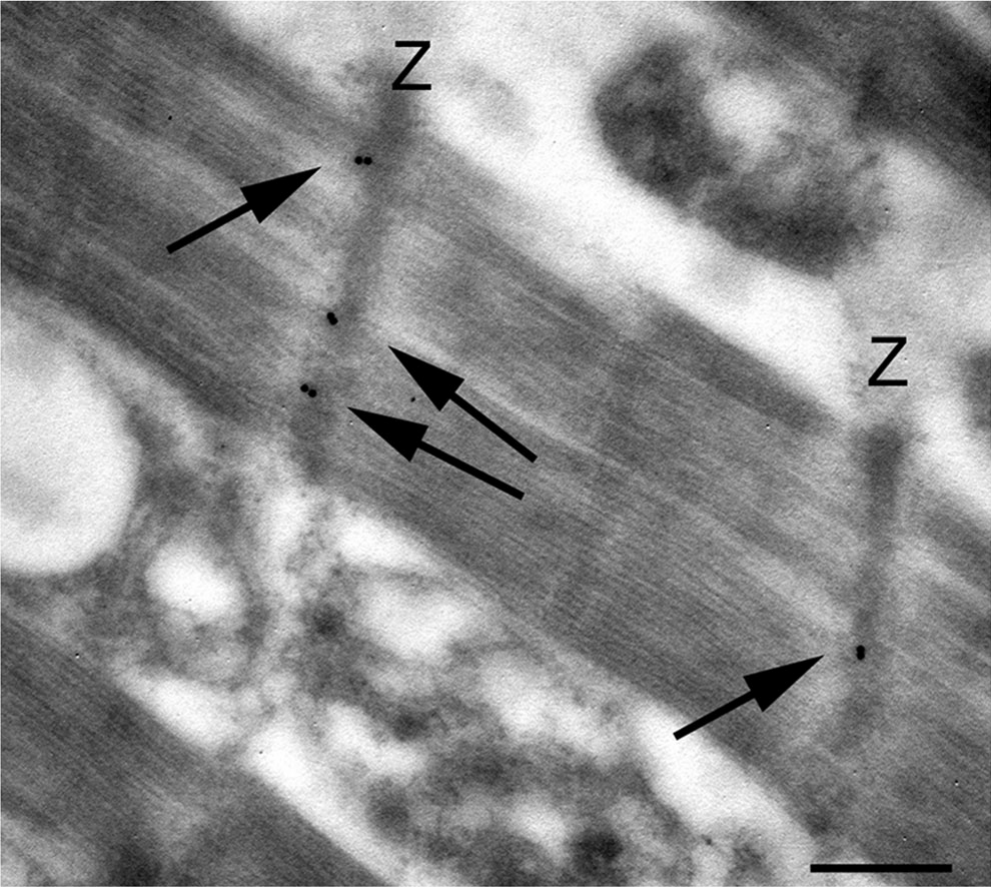
Electron micrographs of myofibrils from human normal myocardium. Immuno-gold localization showing the presence of TG2 (*arrows*) at the level of the Z-band (*Z*). *Bar* 0.3 μm

A large number of TG2 substrates are proteins involved in the organization of the cytoskeleton. Upon activation by Ca^2+^, TG2 contributes to the organization of the cytoskeleton by cross-linking various cytoskeletal proteins, i.e., microtubule protein tau, b-tubulin, actin, myosin, spectrin, thymosin b, troponin T and vimentin (Esposito and Caputo 2005; Kim et al. 2008). TG2 might also regulate contractile performance by means of its function as an adrenergic-receptor-coupled G protein and its important role in stabilizing the cytoskeletal network of developing myotubes has been reported (Bersten et al. 1983). In addition, evidence has been presented for alterations in TG2 expression and function in animals and patients with heart failure (Sane et al. 2007). We examined the cellular distribution of TG2 in myocytes from human heart samples and revealed a particular site for TG2. The enzyme displayed a regular localization along the Z-lines of myofibrils (Fig. 5). Interestingly, in infarct heart failure, myofibrillar disarray and Z band abnormalities are associated with a dislocation of TG2 away from the Z-lines (data not shown).

### TG2 location inside the cell under stressful conditions

**Fig. 6:**
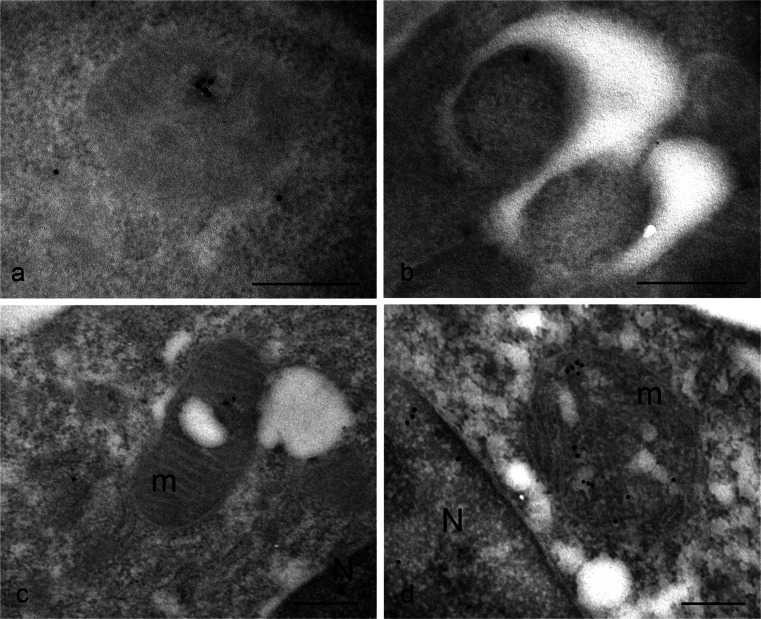
TG2 distribution in HEK293^TG^ cells under stressful conditions. **a**, **b** Under nutrient-deprived conditions, TG2 is found in protein aggregates (**a**) and within autophagosomes (**b**). **c**, **d** Treatment with an uncoupler of oxidative phosphorylation in mitochondrial systems induces clearly visible injury of mitochondria (*m*); under these conditions, immuno-gold detection shows the presence of TG2 inside damaged mitochondria (*N* nucleus). *Bars* 0.3 μm

Two different stress stimuli were used to evaluate the possible modulation of TG2 distribution: starvation and oxidative stress. Under nutrient-deprived conditions, cells responded with a massive induction of autophagy. Under these conditions, immunofluorescence microscopy analysis of p62 and TG2 showed the colocalization of both the proteins inside autophagic vacuoles (Fig. 1i). The immuno-gold analysis demonstrated that, during autophagic induction, TG2 was associated with both protein aggregates (Fig. 6a) and autophagosomes (Fig. 6b).

As is well known, mitochondria undergo dynamic structural alterations to meet changing needs and to maintain homeostasis. Mitochondrial depolarization with carbonyl cyanide m-chlorophenylhydrazone (CCCP) causes severe mitochondrial alterations. Interestingly, TG2 is selectively recruited to damaged mitochondria as demonstrated by the presence of gold granules on mitochondria of cells treated with CCCP (Fig. 6c, d).

### TG2 on the cell surface and extracellular release

Although initially being studied as an intracellular enzyme, TG2 is now known to be secreted into the extracellular space or onto the cell surface (Wang and Griffin 2012). We further investigated the translocation of the enzyme to the cell surface by analyzing pathological conditions known to trigger the extra-cellular release of the enzyme, i.e., celiac disease.

**Fig. 7:**
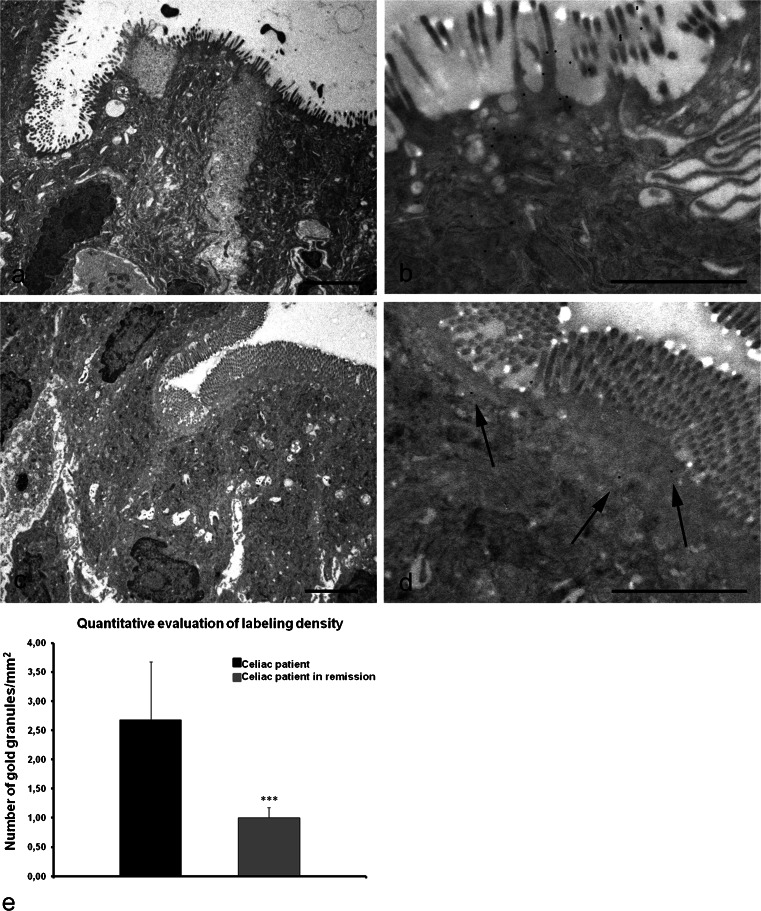
Electron micrographs of duodenal mucosa from celiac patients (**a**, **b**) and from celiac patients on a gluten-free diet (**c**, **d**). **a** Representative example of mucosal injury in a celiac patient: enterocytes display rarefaction and partial disappearance of microvilli. **b** Higher magnification of apical surface showing numerous gold granules: the presence of TG2 appears mostly to be associated with the microvilli on the luminal surface of enterocytes. **c** Ultrastructural features of enteric mucosa in a patient with celiac disease in remission after a gluten-free diet: a marked recovery of tissue architecture is visible, with virtually absent alterations in the brush border. **d** Higher magnification of the top surface facing the lumen: a change in the distribution of TG2 is demonstrated by the presence of just a few granules (*arrows*) localized underneath the microvilli. *Bars* 3 μm (**a**,**c**), 1.5 μm (**b**,**d**). **e** Quantification of TG2 immuno-gold labeling within the apical membranes of enterocytes in celiac patients and in celiac patients on a gluten free-diet. The *bar chart* shows the decrease in the number of gold particles per square micrometer within the apical surface of enterocytes of a patient in remission (*gray*) versus a diseased patient (*black*). *Error bars* are ± SEM. ***P < 0.0001

Despite major advances in understanding the pathogenic role of TG2 in celiac disease, several issues remain to be elucidated, especially the question related to TG2 expression by the enterocytes. The ultrastructural immunolocalization of TG2 in the intestinal epithelium of celiac patients clearly demonstrated an abundant presence of the enzyme at the level of the enterocyte surface, especially on microvilli (Fig. 7b). In samples from celiac patients after a gluten-free diet, the intestinal mucosa displayed remission of the intestinal epithelial damge (Fig. 7c). Quantification of TG2 revealed that the expression of the enzyme on the surface of the enterocytes of these patients was strongly reduced (Fig. 7d, e). The labeling density was 0.3 gold particles/μm^2^ in the biopsies of patients with celiac disease in remission, compared with 2.6 gold particles/μm^2^ in celiac biopsies.

**Fig. 8:**
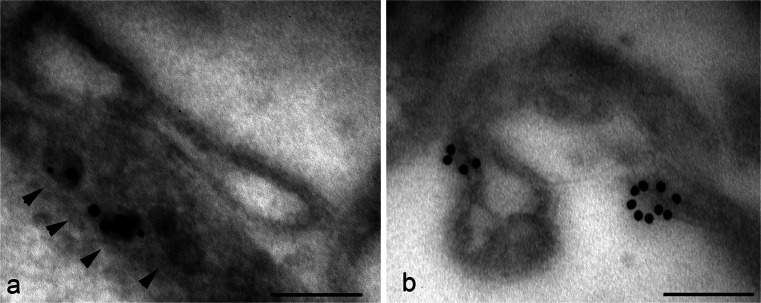
Immuno-gold localization of TG2 in SK-n-BE(2) neuroblastoma cells treated with doxorubicin. The anti-cancer drug doxorubicin causes an extensive distribution of TG2 at the plasma membrane. Gold particles seem to be associated with both dense vesicles (*arrowheads*) located under the cell surface (**a**) and with protruding cell processes (**b**). *Bars* 0.15 μm

A different aspect of TG2 and the extracellular environment relates to cancer cells. TG2 appears to be associated with the early changes in cervical carcinogenesis (Del Nonno et al. 2011) and is involved in epithelial mesenchymal transition, a key step in cancer metastasis (Lin et al. 2011). It has also been reported to be over-expressed in highly aggressive and chemo-resistant human brain, breast, lung and colorectal cancers and to be involved in the migration and invasion of cancer cells such as breast cancer cells and neuroblastomas (Miyoshi et al. 2010; Choi et al. 2011; Oh et al. 2011). Several studies have reported a relationship between TG2 expression and doxorubicin drug resistance but the mechanism has not been fully clarified. Here, the distribution of TG2 was studied in SK-n-BE(2) neuroblastoma cells after doxorubicin treatment (10 μM for 3 h). The immunoelectron microscopic localization showed that treatment with the anti-cancer drug doxorubicin induced a translocation of the enzyme to the cell surface at the level of plasma membrane projections (Fig. 8). Cell surface microvilli displayed numerous microvesicles on their inside; these appeared to be decorated with gold particles indicating the presence of TG2. These observations also provide further insights regarding the possible mechanism utilized by the cell to externalize TG2, suggesting that it is delivered to the surface inside small vesicles (Fig. 8a).

## Discussion

Given the broad function of TG2 in cell growth, survival, death, differentiation, migration and extracellular matrix organization, the cell appears to adapt the dynamics of this enzyme to meet specific sub-cellular needs or to respond to stress or other stimuli. Substantial evidence indicates that the sub-cellular location of TG2 is critical for the regulation of its various biochemical activities, which subsequently trigger diverse downstream events. However, the precise intracellular distribution of TG2 to various sub-cellular compartments remains largely undetermined.

**Fig. 9:**
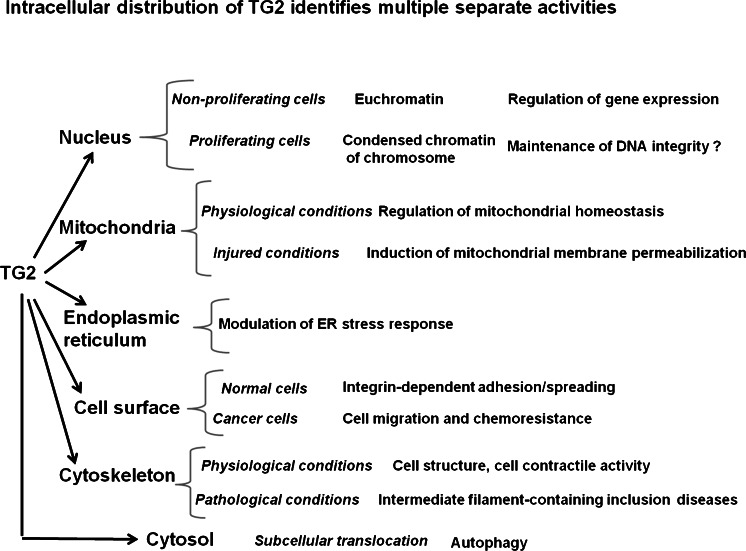
The multifunctional enzyme TG2 is found in various sub-cellular compartments in which its activity is regulated by interaction with diverse factors depending on its location. The identification of changes of location under particular cell conditions might help in the comprehension of unknown functions of TG2

Here, we used an immuno-gold ultrastructural method to establish the fine sub-cellular localization of TG2 under various conditions, either physiological or non-physiological. The data obtained show that TG2 is not freely dispersed in the cell but is always associated with a cell structure and that the presence of TG2 in a cell compartment is not fixed but is determined by the physiological conditions, stress stimuli, or activity state of the cell (Fig. 9).

The presence of TG2 in the nucleus has been reported in various cell types. It generally represents 5–7 % of the total cellular TG2, although this amount can vary depending on cell type, treatment and experimental conditions (Lesort et al. 1998). Nuclear accumulation of TG2 is induced by a number of stressors; in particular, elevated intracellular calcium levels increase the translocation of TG2 to the nucleus (Gundemir et al. 2012). Once in the nucleus, TG2 might mediate transcriptional regulation by modulating the expression of a variety of transcription factors such as nuclear factor kappa Bα and Sp1 and thereby indirectly affect the transcription of a multitude of genes (Mann et al. 2006; Tatsukawa et al. 2009).

Previous data, obtained by immuno-blot on cell fractions, have demonstrated that about 90 % of nuclear TG2 is associated with chromatin and the remaining with nuclear matrix (Lesort et al. 1998). Here, we have revealed that, in cells under non-proliferating conditions, TG2 is not widely distributed within the chromatin but is confined to the euchromatin, the de-condensed chromatin domains required to allow transcription. Given this finding, we do not expect to find TG2 on the chromosome of proliferating cells. Nevertheless, an exact opposite arrangement is observed during mitosis, when TG2 has been detected on highly condensed chromatin. The major change in nuclear structure during mitosis is chromosome condensation. The interphase chromatin, which is packaged into nucleosomes, condenses approximately a further thousand-fold to form the compact chromosomes seen in mitotic cells. Phosphorylation of histone H3 has been found to be required for the condensation of mitotic chromosomes and histone H1 is phosphorylated during mitosis in most cells, even though its phosphorylation seems to be unnecessary (Xu et al. 2009). All four mammalian core histones (H2A, H2B, H3 and H4) have been shown to be glutaminyl substrates of TG2 and their crosslinking contributes to chromatin condensation during the onset of apoptosis (Kim et al. 2002). In addition, histones are also substrates for TG2 kinase activity (Mishra et al. 2006). Biochemical and in vitro experiments have shown that TG2 can phosphorylate H3 and, to a lesser extent, H1. The TG2-induced phosphorylation of H3 occurs at Ser10, a site shown to be crucial for chromosome condensation and cell cycle progression (Nowak and Corces 2004). In recent years, chromatin research has revealed that histones are targets of multiple post-translational modifications, including phosphorylation, acetylation, methylation, ubiquitination and ADP ribosylation. These modifications, which occur on distinct amino acid residues on specific histones and within chromatin at certain genomic regions, make it possible to regulate two opposite processes, such as transcriptional activation and repression, by using the same amino acid sequence within a histone (Cohen et al. 2011). Considering biochemical evidence and the observed presence of TG2 on both euchromatin domains and condensed chromatin, we suggest that TG2 represents one of the effector elements in this network of modifications that control gene expression, chromosome condensation and disease processes.

TG2 appears to exert a double-function by also influencing cell fate at the level of another cell compartment: the mitochondrium. A strong association between oxidative stress and TG2 up-regulation has been reported; this might result in cell survival or apoptosis (Caccamo et al. 2012). We have previously shown that TG2 affects mitochondrial function and regulates the energy balance of the cell (Piacentini et al. 2002). Upon cell death induced by staurosporine treatment, TG2 cross-links Bax on the outer mitochondrial membrane resulting in the stabilization of the Bax oligomer and, consequently, in the opening of pores in the outer mitochondrial membrane and in membrane permeabilization (Rodolfo et al. 2004). In addition TG2 is involved in the regulation of the respiratory chain. Through its PDI activity, the enzyme can stabilize the assembly and activity of some members of the mitochondrial respiratory chain (Malorni et al. 2009). In spite of these data, some authors appear to be sceptical about the localization of TG2 inside mitochondria and are more prone to consider the enzyme as being loosely associated with the outside of the mitochondria (Gundemir et al. 2012). Utilizing the immuno-gold technique, we here clearly show that TG2 can be localized in association with the outer membrane of mitochondria but also inside mitochondria. Of particular interest is the finding that the enzyme is especially recruited to mitochondria that have lost their functional integrity, such as after cell treatment with CCCP, an uncoupling agent that dissipates the mitochondrial membrane potential. These data support the hypothesis that TG2 exerts, at the mitochondrial level, an important role both in physiology and pathology, contributing to set the threshold of cell redox state homeostasis and thus playing a pro-survival or cell-death-inducing function, with special relevance for all those pathologies (neurodegenerative, miocardial dysfunction) in which mitochondrial functionality is of primary importance (Piacentini et al. 2011).

Importantly, our data revealed that TG2 is present on the ER. Several BCL-2 family members have also been found at this location, where they have been reported to be involved in the regulation of calcium homeostasis, autophagy and ER stress responses (Szegezdi et al. 2009). Our results might shed light on other possible functions of TG2 as yet unknown. In this regard, recent work based on a cellular model of Parkinson's disease provided evidence of ER-associated TG2, which the authors propose could have a direct impact on ER function in the pathogenesis of this disease (Verhaar et al. 2012).

In addition to the intracellular functions exerted by TG2, the enzyme is involved in extracellular processes: TG2 mediates cell adhesion in cooperation with fibronectin and the integrins (Verderio et al. 2003; Zemskov et al. 2006) and supports the polymerization of fibronectin and collagen (Jones et al. 1997). As an extracellular protein, TG2 has an established role in celiac disease, both in increasing the immunogenicity of gluten antigens and also as an autoantigen (Klöck et al. 2012). Despite major advances in understanding the pathogenic role of TG2 in celiac disease, several issues remain to be elucidated. These include the mechanism of TG2 externalization and the role of enterocytes as primary sites of gliadin modification by TG2. Immunohistochemical studies have shown that TG2 is expressed in normal small intestine and that such expression is slightly increased in untreated celiac small intestinal mucosa in which TG2 is detected at the level of muscularis mucosae and pericryptal fibroblasts adjacent to enterocytes (Di Sabatino et al. 2012). TG2 expression has also been detected in celiac enterocytes (Biagi et al. 2006). Here, we showed that celiac enterocytes of untreated patients specifically express TG2 on their apical surface and along microvilli facing the intestinal lumen, as opposed to normal control enterocytes that display the presence of positive reaction exclusively at the level of the basolateral membrane. Our observations reveal no evidence for the presence of the enzyme inside the enterocytes, thus supporting the idea that gliadin peptides come into contact with TG2 only outside enterocytes.

Several studies have suggested that extracellular TG2 is also involved in migration of tumor cells by binding to fibronectin or by keratin reorganization (Park et al. 2011; Chen et al. 2010). TG2 can influence several aspects of cancer cells by the activation of survival pathways or the inhibition of apoptosis. In addition, TG2 has been reported to mediate chemo-resistance. The exact mechanism through which TG2 expression mediates drug resistance is not completely understood; however, doxorubicin resistance seems to be dependent upon the action of TG2 on ECM proteins, thereby promoting the interaction between integrins and fibronectin (Herman et al. 2006). In this study, we showed that TG2 is rapidly externalized by neuroblastoma cells after doxorubicin treatment. These results also add information about the process of TG2 externalization. The available data in the literature concerning mechanisms of TG2 translocation to the plasma membrane suggest at least two possible routes: the formation of microparticles ranging between 500 and 1000 nm in size (Van den Akker et al. 2012) or the involvement of an endosomal recycling pathway (Zemskov et al. 2011). Our immuno-gold analysis revealed that doxorubicin induces the externalization of TG2 by utilizing small vesicles (around 50–100 nm) apparently formed under the plasma membrane and perhaps originating from the ER, as suggested by double-staining with calreticulin (data not shown). These data agree with published studies showing the involvement of TG2 in the regulation of exocytosis or neurotransmitter release (Pastuszko et al. 1986; Gobbi et al. 1996; Santhanam et al. 2011) and will require further investigation.

A number of studies have demonstrated that TG2, in addition to its role on ECM scaffolds, can affect cell plasticity. In agreement with this, TG2 has recently been established to be able to mediate axonal microtubules stability by the polyamination of tubulins (Song et al. 2013). By contrast, TG2 can regulate the cell contractile response by modulating F-actin polymerization and myosin light chain phosphorylation (Spurlin et al. 2009). TG2 is widely expressed by cardiac and vascular cells including endothelial and smooth muscle cells, which constitutively express TG2 at high levels. Cardiomyocytes from adult hearts also express TG2. Evidence exists for alterations in TG2 expression and function in animals and patients with heart failure. TG2 is up-regulated during cardiac hypertrophy and heart failure in animal models, whereas its activity (GTP binding and GTPase) has been found to be down-regulated in patients with ischemic and dilated cardiomyopathies (Sane et al. 2007). Here, we provide, for the first time, evidence for the specific location of TG2 in cardiac myocytes Z-disc. The Z-disc is a critical element in the regulation of myocardial function by changes in the cell stresses and strains that accompany altered hemodynamic demands. Evidence of an association between cardiac Z-discs and signaling proteins such as kinases, phosphatases and Ca^2+^-binding proteins indicates an important but poorly understood potential for Z-disc involvement in the development of myocardial hypertrophy, myopathies and heart failure (Pyle and Solaro 2004). Our findings suggest that TG2 is part of the network of proteins making up the Z-disc; it might be active in the stabilization of structural proteins or function as a signaling protein. According to this point of view, the biochemical and in vitro data in the literature indicate a strong noncovalent interaction between the transglutaminase molecule and the myofibril Z line (Gard and Lazarides 1979). Although the details of the interaction between TG2 and cardiac Z-discs are unknown, our finding of the dislodgment of TG2 in myofibrils during heart failure appears intriguing.

The accurate function of proteins and their interaction networks is heavily dependent on the proper localization of each protein. Knowing the location of a protein within its cellular environment is critical for an understanding of the regulatory mechanisms by which it is controlled. With the utilization of immunoelectron microscopy as an experimental tool, this study provides direct information concerning TG2 cellular and sub-cellular localization. Even if other methodological approaches have been adopted to date to define TG2 locations, electron microscopy remains the most accurate technique for demonstrating the relationship between protein distribution and morphological events. In this study, we also described a possible unrecognized location of TG2. Our findings thus provide useful insights about the functions and regulation of this pleiotropic enzyme.
